# Relaxation Effect of Nature Sound Exposure on Gambling Disorder Patients: A Crossover Study

**DOI:** 10.1089/jicm.2022.0611

**Published:** 2023-08-09

**Authors:** Hiroko Ochiai, Harumi Ikei, Hyunju Jo, Masayuki Ohishi, Yoshifumi Miyazaki

**Affiliations:** ^1^ Department of Plastic and Reconstructive Surgery, National Hospital Organization Tokyo Medical Center, Meguro-ku, Japan.; ^2^ Center for Environment, Health and Field Sciences, Chiba University, Kashiwa, Japan.; ^3^ Ohishi Clinic, Yokohama, Japan.

**Keywords:** nature therapy, gambling disorder, near-infrared spectroscopy, prefrontal cortex activity, physiological relaxation, profile of mood states

## Abstract

**Objective:**

Gambling disorder (GD) has been associated with economic, social, mental, and physical problems. Alternative leisure activities or stress-relieving activities have been adopted as part of GD treatment. Moreover, it has been proven that activities utilizing the natural environment, such as shinrin-yoku, have a relaxing effect on healthy people. In this study, we examined the physiological and psychological responses of patients with GD to determine whether nature therapy could reduce their stress responses.

**Design:**

This study included 22 Japanese male participants who were found to be pathological gamblers, with a South Oaks Gambling Screen score of ≤5. We exposed the participants to the digital nature sounds of insects and city sounds of a scramble intersection. The nature and city sounds were presented in a counterbalanced order.

**Outcome measures:**

A two-channel near-infrared spectroscopy system was used to measure the changes in oxyhemoglobin (oxy-Hb) concentrations in the bilateral prefrontal cortex. The heart rate variability was measured to evaluate the autonomic nervous activity. Subjective evaluation was performed using the modified version of the semantic differential method and the Profiles of Mood States, Second Edition (POMS2).

**Results:**

The oxy-Hb level in the bilateral prefrontal cortex significantly decreased. No significant difference in the high-frequency (HF) and low-frequency/HF ratio was observed. The subjective evaluation indicated that the participants experienced increased comfort and relaxation and had more natural feelings. Nature sounds significantly decreased the POMS2 negative emotion subscale and total mood disturbance scores and increased the positive emotion subscale scores. Nature-based stimulus exposure induces physiological relaxation and other positive effects among individuals even with GD.

**Conclusion:**

Exposure to nature-based sounds induces physiological relaxation and other positive responses among individuals with GD. In patients with GD, nature sounds produce the same relaxation response as in healthy individuals. (Umin.ac.jp under registration number: UMIN000042368).

## Introduction

Gambling addiction is officially referred to as “pathological gambling” by the World Health Organization and “gambling disorder” by the Diagnostic and Statistical Manual of Mental Disorders, Fifth Edition (DSM-5).^1,2^ Stress has been implicated as one of the causes of development and relapse of gambling disorder (GD).^3^ The underlying mechanism of addiction is that the neuronal network of interacting brain regions and associated circuits is disrupted. Therefore, the method of reducing the reactivity of stress-associated circuits by the use of biofeedback or medications has been proposed for the treatment of GD.^4^ Cognitive behavioral therapy (CBT) is also recognized as one of the effective treatments for GD, which is commonly discussed in meta-analysis reviews.^5^ To date, no absolute treatment method (e.g., CBT^6^) or program for GD is available.

This study considered the use of nature therapy as one method that meets the criteria for GD treatment, which is an effective and convenient way to alleviate stress, with some meta-analyses having shown that all psychotherapies are effective in some way,^7^ for example, it has been reported that participating in alternative leisure activities, particularly inexpensive, pleasurable, or social activities, as a replacement for gambling behavior may decrease the likelihood of GD.^8^

With regard to the use of the natural environment, it is recommended that the human body be exposed to the natural environment^9^ because most people worldwide have few opportunities to interact with nature daily,^10,11^ which is associated with increased isolation and depression.^12,13^ Based on physical and psychological data,^14,15^ forest bathing, also known as “shinrin-yoku,” has been found to be advantageous as it can help one experience the complex and diverse elements of the ecosystem and synchronize with nature.^16–20^

Furthermore, several scientific studies have shown that physiological relaxation and stress recovery can be achieved by contact with nature through the five senses even indoors.^21–24^ It has also been reported that exposure to auditory stimulation of a murmuring brook induced physiological and psychological relaxation, resulting in decreased oxyhemoglobin (oxy-Hb) concentrations in the prefrontal cortex activity and decreased sympathetic nervous activity in healthy subjects.^25^ Based on these findings, we have previously reported that the sound of a murmuring creek improved the prefrontal cortex activity in people with GD.^26^ However, the number of participants in our report was too small.

The aim of this study is to increase the number of subjects and to clarify the physiological and psychological relaxation effects of insect sounds, one of the typical nature sounds, as a stimulus on GD.

## Materials and Methods

### Research design

A within-subjects experiment was conducted at the Ohishi Clinic (psychiatric clinic) with patients diagnosed and being treated for GD by a psychiatrist. The experiment protocol was approved by the ethics committee of the Center for Environment, Health and Field Science, Chiba University (project identification number 49). The study conformed to the Declaration of Helsinki, and it was registered in the University Hospital Medical Information Network of Japan (UMIN ID: UMIN000042368, https://center6.umin.ac.jp/cgi-open-bin/ctr_e/ctr_view.cgi?recptno=R000048365) before participant inclusion. Experimental procedures, physiological and psychological measurements, and statistical analyses were performed, as described in a previous study.^26^

### Participants

Patients who were diagnosed with GD by a psychiatrist and who were treated for psychiatric impairment, as well as those without respiratory diseases, such as asthma and chronic rhinitis, were recruited. In line with our previous research on the physiological relaxing effects of nature,^21–25^ the target sample size for this study was set to 15–30; participant recruitment was conducted in early October 2020. Before this experiment, the 25 participants were gathered in a waiting room at the Ohishi Clinic (psychiatric clinic) and were briefed on the purpose and methods of this experiment in detail.

**FIG. 1.**
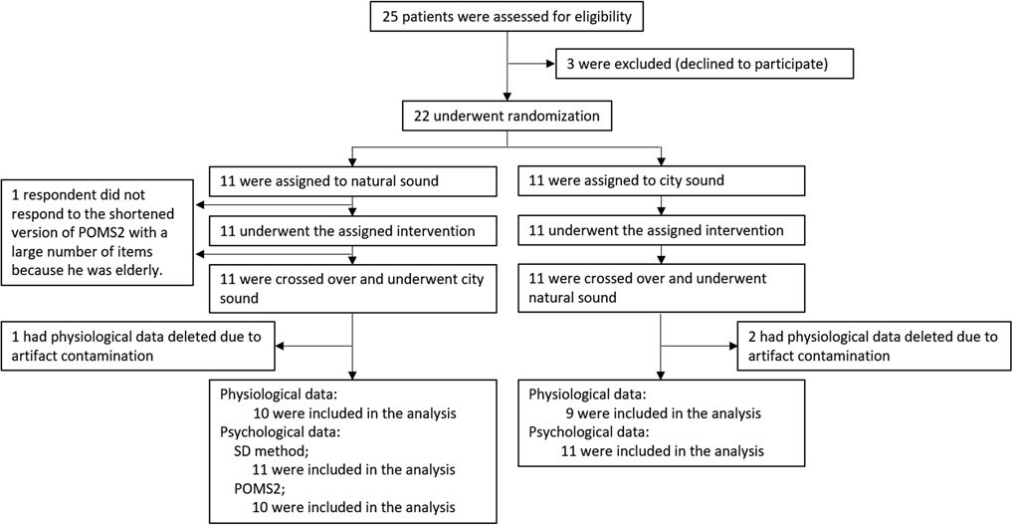
Flowchart of the experiment based on the CONSORT statement (checklist in Supplementary Table S1). Subject screening, enrollment, follow-up, and analysis flow.

Those who agreed to participate provided a written informed consent form. Next, the participants were instructed to complete the modified Japanese version of the South Oaks Gambling Screen (SOGS),^27^ which is a modified version of the GD evaluation scale with scores of 0–20. Patients with a total score of 5 or higher are diagnosed with GD. Moreover, individuals diagnosed with GD (mean score, 13.0 ± 2.8; min, 6 points; max, 16) were included in this study. Figure 1 shows a flowchart of the experiment.

### Auditory stimulation

The participants used an MP3 to listen to sounds. For auditory stimulation using nature sounds, the insect sounds were played at 49.5 dB, which were purchased from Audiostock. This sound was recorded at about 3:00 a.m. at a riverside in Osaka in autumn. For auditory stimulation using city sounds, we used the sound of traffic at an intersection in Shibuya, Tokyo. We played the sound of a scramble intersection at 49.4 dB downloaded from the Sound Effects Lab website. Two sets of headphones (Audio-Technica ATH-M20x) were prepared. Hence, each participant could use one set of headphones. Two participants used one audio player at the same time with a two-way distribution cable. Therefore, the order of stimulus presentation was counterbalanced for each pair.

### Study protocol

The physiological experiment was conducted in an isolated room of Ohishi Clinic for 3 days. The mean temperature, relative humidity, and illumination of the indoor environment for 3 days were 23.4°C ± 1.5°C (mean ± standard deviation [SD], hereafter the same), 46% ± 6%, and 150 ± 70 lx at eye level, respectively.

**FIG. 2.**
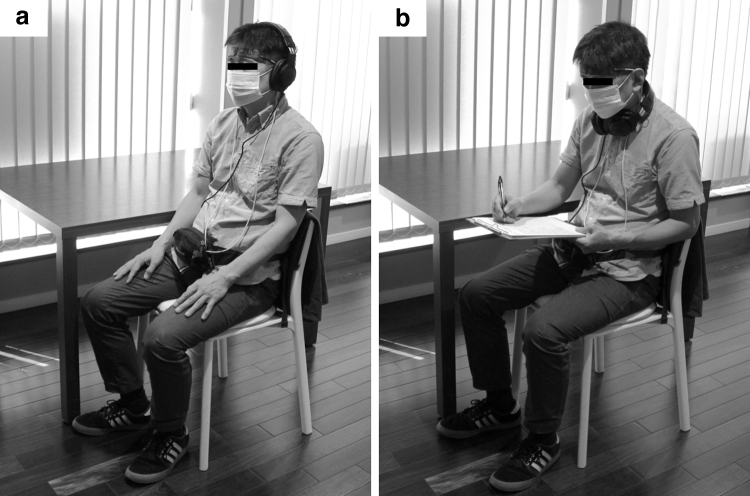
Measurement scene. **(a)** During visual stimulation; **(b)** fill out the questionnaires.

Sensors for heart rate variability (HRV) were attached to the participants. Then, two participants entered the room each time and sat on their assigned chairs. An HRV measurement device, near-infrared spectroscopy (NIRS) system, and sound-producing headphone were attached to the participants (Fig. 2a). After the measurement procedures were briefed, the two participants simultaneously received auditory stimuli, while sitting down with eyes closed. In the actual experiment, the participants first practiced using a dummy sound source (the sound of ocean ripples) using the same procedure. Afterward, the actual experiment was conducted.

First, the participants put on the headphones and maintained in a resting state, while in sitting position with eyes closed for about 2 min. The experimenter turned on the audio player and played the auditory stimuli for nature or city sounds for 60 sec. Next, the participants answered the subjective evaluation questionnaire (Fig. 2b). Physiological activity was measured continuously between the resting state and the auditory stimulation. Moreover, psychological activity was measured using the modified version of the method and the Profiles of Mood States, Second Edition (POMS2), for ∼2 min.

### Physiological measurements

#### Near-infrared spectroscopy

A two-channel NIRS system (PocketNIRS Duo; DynaSense, Shizuoka, Japan) was used to measure changes in the oxy-Hb concentrations in the bilateral prefrontal cortex.^28^ The NIRS probes were placed symmetrically on the bilateral foreheads of the participants, while the participants were resting and receiving auditory stimuli, and the changes in oxy-Hb concentrations in the bilateral prefrontal cortex were recorded at 1-sec intervals. The value for 10 sec before stimulus onset was set to 0, and subsequent changes were recorded for 60 sec.

#### Heart rate variability and heart rate

HRV and heart rate (HR) were used as autonomic nervous system (ANS) activity parameters^29^ and were evaluated using a portable electrocardiograph (Activtracer AC-301A; GMS, Tokyo, Japan). Power levels with a range of 0.15–0.40 Hz for the high-frequency (HF) component and 0.04–0.15 Hz for the low-frequency (LF) component of HRV were analyzed (MemCalc/win; GMS).^30^ HRV recordings were analyzed for 60 sec before and during stimulation to investigate the acute physiological responses to sound stimuli.

### Psychological measurements

The emotions caused by each auditory stimulus were assessed using a modified version of the SD method and the POMS2. A 13-point scale between three sets of indices (comfortable to uncomfortable, relaxed to awakening, and natural to artificial), which is a modified version of the SD method, was used.^31,32^ The POMS2 score was created with the following subscales: anger–hostility (A–H),; confusion–bewilderment (C–B); depression–dejection (D–D); fatigue–inertia (F–I); tension–anxiety (T–A); vigor–activity (V–A); and friendliness (F). To reduce the participants' efforts, the POMS2 was simplified with only 35 questions.^33^ The total mood disturbance (TMD) score was calculated using the following formula: (A–H) + (C–B) + (D–D) + (F–I) + (T–A) − (V–A). When the TMD score was higher, the psychological state was worse.

### Statistical analyses

To validate the physiological and psychological effects of auditory stimulation using nature and city sounds, we compared the physiological and psychological responses to nature and city sounds during auditory stimulation by paired t-test and Wilcoxon signed-rank test, respectively. In addition, as a subanalysis, we compared the physiological status before (NIRS, 10 sec; HRV and HR, 60 sec) and during the stimulation (60 sec) of nature sounds. The Statistical Package for the Social Sciences software version 25.0 (IBM, NY) was used, and a *p*-value of <0.05 was considered statistically significant.

## Results

### Participants

In total, 22 participants who were classified as pathological gamblers with a SOGS score of ≤5 were included in the experiment. The participants were men 25–60 years of age (mean age ± SD: 40.0 ± 9.4 years). They were randomly assigned to two study groups, as shown in Figure 1 (11 participants in each group). One participant and three participants with no data from the POMS2 and NIRS, respectively, were excluded from the analysis.

### Physiological effects

#### Near-infrared spectroscopy

**FIG. 3.**
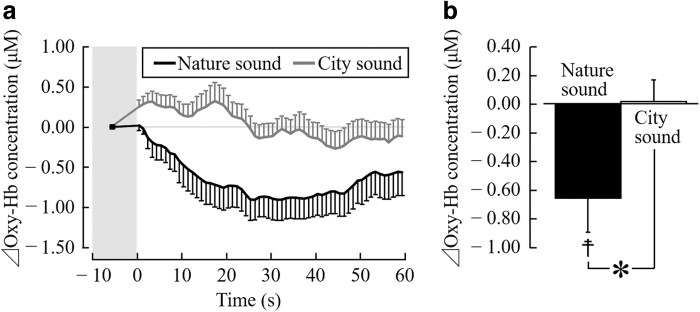
oxy-Hb concentration in the *left* prefrontal cortex during auditory stimulation with nature and city sounds. **(a)** Change in each 1-sec average oxy-Hb concentration over 60 sec of stimulation (difference from the mean value for 10 sec before stimulation). **(b)** Overall mean oxy-Hb concentration. Data are expressed as the mean ± SE, *n* = 19, *p < 0.05 (comparing nature vs. city sounds), ^☨^*p* < 0.05 (comparing premeasurement vs. postmeasurement value), as determined by the paired *t*-test. oxy-Hb, oxyhemoglobin; SE, standard error.

Figure 3a shows the changes in the oxy-Hb concentrations in the left prefrontal cortex every 1 sec, while listening to nature and city sounds. The oxy-Hb concentrations in the left prefrontal cortex decreased immediately after the start of stimulation using nature sounds and reached the minimum after 26–47 sec. On the contrary, the oxy-Hb concentrations in the prefrontal cortex increased immediately after the start of stimulation using city sounds, and gradually decreased after 11 sec.

Figure 3b shows the overall mean oxy-Hb concentration in the left prefrontal cortex during the 1-min auditory stimulation period. Compared with listening to city sounds for 1 min, the average oxy-Hb concentration in the left prefrontal cortex significantly reduced after listening to nature sounds (nature sounds, −0.65 ± 0.24 μM and city sounds, 0.01 ± 0.15 μM; *t*(18) = 2.29; *p* = 0.034; Fig. 3b). Therefore, the oxy-Hb concentrations significantly decreased after listening to nature sounds (*t*(18) = 2.78; *p* = 0.012; Fig. 3b).

**FIG. 4.**
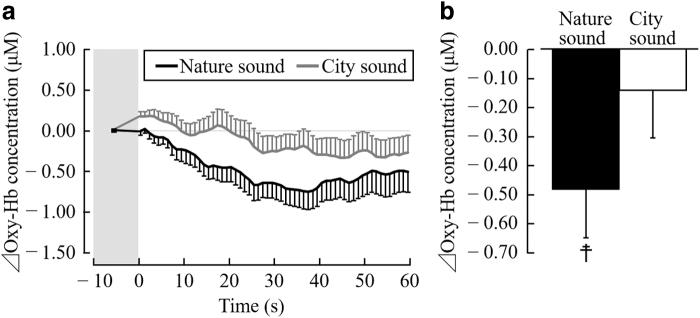
oxy-Hb concentration in *right* prefrontal cortex during auditory stimulation with nature and city sounds. **(a)** Change in each 1-sec average oxy-Hb concentration over 60 sec of stimulation (difference from the mean value for 10 sec before stimulation). **(b)** Overall mean oxy-Hb concentration. Data are expressed as the mean ± SE, *n* = 19, ^☨^*p* < 0.05 (comparing premeasurement vs. postmeasurement value), as determined by the paired *t*-test. oxy-Hb, oxyhemoglobin; SE, standard error.

Figure 4 shows the changes in the oxy-Hb concentrations in the right prefrontal cortex. The oxy-Hb concentration in the right prefrontal cortex decreased immediately after the start of stimulation using nature sounds and reached the minimum after 33–40 sec. Moreover, the oxy-Hb concentrations in the prefrontal cortex increased immediately after the start of stimulation using city sounds and gradually decreased and stabilized after the 25-sec time point (Fig. 4a).

Figure 4b shows the overall mean oxy-Hb concentration in the right prefrontal cortex during the 1-min auditory stimulation period. While the participants were listening to nature and city sounds, the oxy-Hb concentrations in the right prefrontal cortex were −0.48 ± 0.17 and −0.14 ± 0.16 μM, respectively, whereas the oxy-Hb concentration in the right prefrontal cortex significantly decreased after listening to nature sounds (*t*(18) = 2.92; *p* = 0.009; Fig. 4b). However, no significant difference in the average oxy-Hb concentrations in the right prefrontal cortex after listening to nature and city sounds was observed (*t*(18) = 1.53; *p* = 0.142; Fig. 4b).

#### HRV and HR

The HF and LF/HF ratio did not significantly differ after 1-min auditory stimulation using nature and city sounds.

### Psychological effects

**FIG. 5.**
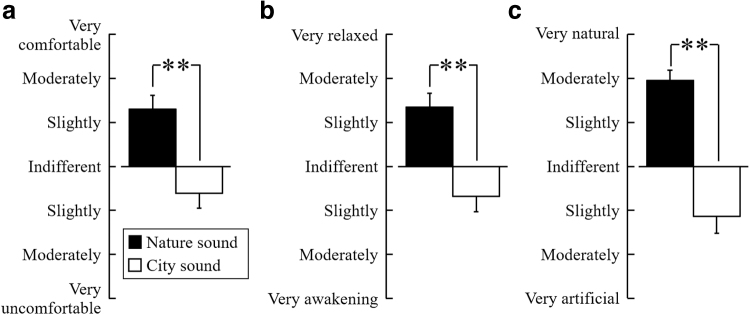
Subjective evaluation of participants based on the modified semantic differential method questionnaire. The questionnaire was given to the participants after listening to nature or city sounds. **(a)** Comfortable feeling; **(b)** relaxed feeling; **(c)** natural feeling. Data are expressed as the mean ± SE, *n* = 22, **p < 0.01 (comparing nature vs. city sounds), as determined by the Wilcoxon signed-rank test. SE, standard error.

Figure 5 shows the results of the modified version of the SD method. Based on the subjective evaluation, the participants had increased comfort (*p* < 0.01) (Fig. 5a) and relaxation (*p* < 0.01) (Fig. 5b) and more natural feelings (*p* < 0.01) (Fig. 5c) after listening to nature sounds compared with city sounds.

**FIG. 6.**
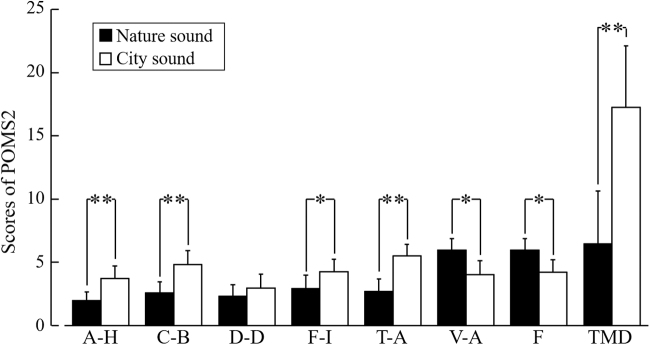
Subjective feelings based on the POMS2. The seven subscales of the POMS2 were assessed after listening to nature or city sounds. A–H, anger–hostility; C–B, confusion–bewilderment; D–D, depression–dejection; F–I, fatigue–inertia; T–A, tension–anxiety; V–A, vigor–activity; F, friendliness. The TMD score was calculated using the following formula: ([T–A] + [D–D] + [A–H] + [F–I] + [C–B] − [V–A]). Data are expressed as the mean ± SE, *n* = 21, **p < 0.01, and *p < 0.05 (comparing nature vs. city sounds), as determined by the Wilcoxon signed-rank test. POMS2, Profiles of Mood States, Second Edition; SE, standard error; TMD, total mood disturbance.

Figure 6 shows the results of the POMS2 assessment. After listening to nature sounds, the scores for the negative subscales, including A–H (*p* < 0.01), C–B (*p* < 0.01), F–I (*p* < 0.05), and T–A (*p* < 0.01), significantly decreased. However, the scores for V–A (*p* < 0.05) and F (*p* < 0.05), which are positive emotions, increased significantly after listening to nature sounds. The TMD score reduced significantly after listening to nature sounds (*p* < 0.01).

## Discussion

This study assessed the effects of listening to nature- and city-based sounds among individuals with GD. The participants' responses to auditory stimuli were investigated under laboratory conditions. In this research, we reduced the elements of the natural environment to a single stimulus to simplify the evaluation. We focused on the sound of buzzing insects, which is one of the natural environmental elements. Then, the reaction of participants with GD to this sound alone was evaluated. The following results were obtained: (1) listening to nature sounds significantly reduced the oxy-Hb concentrations in the bilateral prefrontal cortex. (2) The results of the assessment using the modified SD method showed that nature-based stimuli significantly increased comfort, relaxation, and natural feelings. (3) The POMS2 negative subscale scores significantly decreased after listening to nature sounds compared with city sounds.

Song et al. showed that various nature therapies had physiological relaxation effects on activities in the central nervous system, ANS, endocrine system, and immune system, from the perspective of EBM.^14^ The results of our experiment are consistent with those of previous studies with regard to the physiological and psychological responses to naturally occurring stimuli in healthy participants.^34–36^

Furthermore, our results are also consistent with those of previous reports on the physiological and psychological relaxation effects of nature-based auditory stimuli on patients with GD.^26^ In our previous report, we investigated the effects of a nature-derived auditory stimulation using the murmuring creek sound on 12 male patients with GD and found that the oxy-Hb levels of the left and right prefrontal cortices were significantly decreased, resulting in a relaxed state of being. In this study, the number of subjects was increased to 22, and the stimulus sound was changed from “murmuring creek” to “insect sounds.” The results indicated that, despite the change in the types of sound, nature sounds could calm the prefrontal cortex activities and produce the same relaxation effect.

From the perspective of neurotransmitters, an inseparable relationship between stress and the risk of developing addiction can be observed,^37^ and daily stress could be associated with the urge to gamble.^38^ High levels of emotional stress can lead to loss of impulse control and inability to control inappropriate behavior and tolerate frustration.^39,40^ It has also been reported that people are spending more time at home and are in a state of stress related to the COVID-19 pandemic. While notably this study showed a physiologically significant relaxation response to nature sounds in individuals with GD as well as in healthy participants,^25^ these circumstances resulted in a demand for familiar relaxation methods, and the results of this experiment suggest that the auditory stimulation of nature-derived sounds may be useful for patients with GD.

However, several limitations must be considered when interpreting the results of this study. First, a risk of selection bias is present because the participants had stable symptoms based on the assessment performed by psychiatrists. Second, we limited our experiments to men as GD tends to be predominant in men. Thus, further studies should be conducted to examine the relationship between impulsivity and physiological stress responses among individuals with GD, especially women.

Third, the exposure time to the stimuli was short. Since we compared the relaxation levels before and 1 min after exposure to stimulation using nature and city sounds, the results were only preliminary. Fourth, we did not check the extent of participants' contact with nature. The frequency of contact with nature could influence the results. The relationship between specific stimuli and biological responses is a complex research topic that is challenging to investigate. Therefore, more extensive research about the appropriate extent, duration, and frequency of stimulus exposure and types of stimuli should be conducted.

## Conclusions

The changes in physiological and psychological responses among patients with GD who were exposed to nature and city sounds were examined. This study revealed the following results: (1) listening to nature sounds significantly decreased the oxy-Hb concentrations in the bilateral prefrontal cortex, thereby promoting a pleasant and relaxed state; (2) based on the assessment using the modified SD method, nature-related stimuli significantly increased comfort, relaxation, and natural feelings; and (3) stimulation with nature sounds significantly decreased the A–H, C–B, F–I, T–A, and TMD subscale scores. However, the V–A and F subscale scores increased remarkably. Exposure to nature sounds showed physiological relaxation and other positive responses among individuals with GD. Thus, nature sounds have a stress-reducing effect on patients with GD, as well as healthy individuals.

## Supplementary Material

Supplemental data
